# Combining Phylogeography with Distribution Modeling: Multiple Pleistocene Range Expansions in a Parthenogenetic Gecko from the Australian Arid Zone

**DOI:** 10.1371/journal.pone.0000760

**Published:** 2007-08-22

**Authors:** Jared L. Strasburg, Michael Kearney, Craig Moritz, Alan R. Templeton

**Affiliations:** 1 Department of Biology, Washington University, St. Louis, Missouri, United States of America; 2 Department of Zoology and Centre for Environmental Stress and Adaptation Research, The University of Melbourne, Parkville, Victoria, Australia; 3 Museum of Vertebrate Zoology, University of California at Berkeley, Berkeley, California, United States of America; Fred Hutchinson Cancer Research Center, United States of America

## Abstract

Phylogenetic and geographic evidence suggest that many parthenogenetic organisms have evolved recently and have spread rapidly. These patterns play a critical role in our understanding of the relative merits of sexual versus asexual reproductive modes, yet their interpretation is often hampered by a lack of detail. Here we present a detailed phylogeographic study of a vertebrate parthenogen, the Australian gecko *Heteronotia binoei*, in combination with statistical and biophysical modeling of its distribution during the last glacial maximum. Parthenogenetic *H. binoei* occur in the Australian arid zone and have the widest range of any known vertebrate parthenogen. They are broadly sympatric with their sexual counterparts, from which they arose via hybridization. We have applied nested clade phylogeographic, effective migration, and mismatch distribution analyses to mitochondrial DNA (mtDNA) sequences obtained for 319 individuals sampled throughout the known geographic ranges of two parthenogenetic mitochondrial lineages. These analyses provide strong evidence for past range expansion events from west to east across the arid zone, and for continuing eastward range expansion. Parthenogen formation and range expansion events date to the late Pleistocene, with one lineage expanding from the northwest of its present range around 240,000 years ago and the second lineage expanding from the far west around 70,000 years ago. Statistical and biophysical distribution models support these inferences of recent range expansion, with suitable climatic conditions during the last glacial maximum most likely limited to parts of the arid zone north and west of much of the current ranges of these lineages. Combination of phylogeographic analyses and distribution modeling allowed considerably stronger inferences of the history of this complex than either would in isolation, illustrating the power of combining complementary analytical approaches.

## Introduction

All vertebrate parthenogenetic lineages examined in any detail have been found to be quite young in evolutionary terms, typically being no more than one million years old and often much younger [1]. Recent origins are also suggested by the ‘twiggy’ taxonomic distribution of parthenogenetic organisms [2]–[4], which are taxonomically widespread but extremely ‘species’ poor within any given lineage [with very few exceptions–see 5]. Despite the apparently limited life-spans of most parthenogenetic lineages, they can potentially be very successful in the short term, as evidenced by their often broad geographic distributions and by molecular signatures of rapid range expansions [6]–[8]. Considerable effort has gone into explaining these patterns and their implications for the importance of sexual reproduction in evolution [4], [9]–[12], but interpretations have often been hampered by a lack of detailed phylogeographic data.

To properly understand the evolutionary dynamics of parthenogenesis, it is necessary to compare the amount and distribution of genetic variation in parthenogenetic lineages with that in closely related sexual lineages [1]. This can allow the identification of parental taxa [13] as well as provide information on the number of clonal origins [14], the ages of clonal lineages [15], and the proportion of genetic variation in parthenogens due to post-formation mutation [16]. Recently developed molecular markers and analytical techniques have allowed for more detailed and informative genetic and phylogeographic comparisons between sexual and asexual taxa [7], [17]–[19]. In addition, combination of phylogeographic approaches with analyses of ecological tolerances and interactions can permit cross-validation of phylogeographic inferences [20] and lead to considerably more insight into the underlying processes that generate the observed patterns of geographic distributions, amounts and distributions of genetic variation, and ecological and climatic correlates [e.g. 21], [22], [23].

Here we present a detailed phylogeographic analysis of parthenogenesis in a vertebrate, the Australian gecko *Heteronotia binoei*. We then combine this with high-resolution statistical [24] and biophysical [25] distribution models to make inferences of their likely distributions during the last glacial maximum (LGM). Parthenogenetic *H. binoei* have the largest range of any known vertebrate parthenogen, including extensive areas where they overlap with the ranges of their sexual counterparts. These attributes make them an appealing subject for the study of adaptation and evolutionary success in parthenogens, and of interactions between parthenogens and their parental taxa. *Heteronotia binoei* is a complex of several diploid sexual chromosome races and two mitochondrially distinct lineages of triploid parthenogenetic clones that formed via hybridization between two of the sexual chromosome races [26]. The CA6 and SM6 sexual chromosome races were involved in reciprocal hybridization events giving rise to the 3N1 and 3N2 (so named because they are triploid) parthenogenetic mtDNA lineages [27]. A third sexual chromosome race, EA6, was not involved in the hybridization events but is geographically widespread and sympatric with 3N1 parthenogens in part of its range. Numerous other sexual chromosome races are much more geographically localized and not as well characterized [26]. Parthenogenetic *H. binoei* exhibit substantial nuclear genetic diversity within each mtDNA lineage, mostly as a result of repeated backcrossing events between putative diploid female hybrids and sexual males [16].

Considerable work has been done characterizing the sexual and parthenogenetic taxa using cytology [28], allozymes [16], and mtDNA restriction profiles [6]. Our recent detailed phylogeographic study of the three widespread sexual races, including the two involved in the hybridization events [29], indicates that they diversified approximately 6 million years ago and expanded into the Australian arid zone during an extended period of gradual aridification throughout much of continental Australia. We have also presented molecular and distributional evidence that *H. binoei* and an invertebrate from the Australian arid zone, the grasshopper *Warramaba virgo*, have evolved hybrid parthenogenesis in parallel and in a strikingly similar fashion, both geographically and temporally [30]. Here we extend this work with an analysis of the origin, spread, and current population structure of parthenogenetic *H. binoei* using more powerful molecular markers and coalescent-based population genetic techniques. We consider the formation and expansion of the parthenogenetic lineages within the context of the last few glacial cycles, in which glacial intervals in much of continental Australia have been associated with increased aridity [31]. In addition, we compare our results to statistical [24] and biophysical [25] distribution analyses of the *H. binoei* complex, and extend these analyses to consider climatic conditions during the LGM. Our combined analyses allow for robust descriptions of the formation and expansion of *H. binoei* parthenogenetic lineages during the last two glacial cycles, and they suggest further avenues of research into the evolutionary dynamics of this complex.

## Results

DNA sequences for all parthenogens ranged from 1283 to 1286 bases. Sequences were aligned manually, and at eight places gaps of one to two bases were inserted to keep all sequences in alignment. All indels occurred within or between adjacent tRNA genes. The aligned DNA sequences consisted of 1289 characters. Summary sequence diversity data for each lineage and for regions within lineages are shown in Table 1.

**Table 1:** Summary sequence information for each mtDNA lineage.

Lineage/Region	N	# Haplotypes	% Nuc Div (SE)	Nesting Clades
3N1 Total	230	43	0.170 (0.065)	
3N1 East Central	48	12	0.085 (0.040)	1-1, 1-2, 1-4 (2-1, 2-2)
3N1 Far West	17	2	0.034 (0.025)	1-4, 1-5 (2-2)
3N1 Northeast	26	4	0.029 (0.018)	1-1, 1-2 (2-1)
3N1 Northwest	10	2	0.047 (0.027)	1-2, 1-4 (2-1, 2-2)
3N1 Southeast	21	3	0.104 (0.057)	1-1, 1-2, 1-4 (2-1, 2-2)
3N1 Southwest	55	16	0.078 (0.029)	1-3, 1-4, 1-6, 1-8, 1-9 (2-2)
3N1 West Central	52	13	0.062 (0.027)	1-4, 1-7 (2-2)
3N2 Total	89	16	0.135 (0.050)	
3N2 Central	27	4	0.090 (0.051)	1-2, 1-3, 1-6 (2-1, 2-3)
3N2 Far West	22	6	0.078 (0.037)	1-2, 1-3 (2-1)
3N2 Northeast	15	7	0.203 (0.067)	1-4, 1-5, 1-6, 1-7 (2-2, 2-3)
3N2 Southeast	24	4	0.110 (0.055)	1-1, 1-2, 1-3, 1-6 (2-1, 2-3)

**Figure 1:**
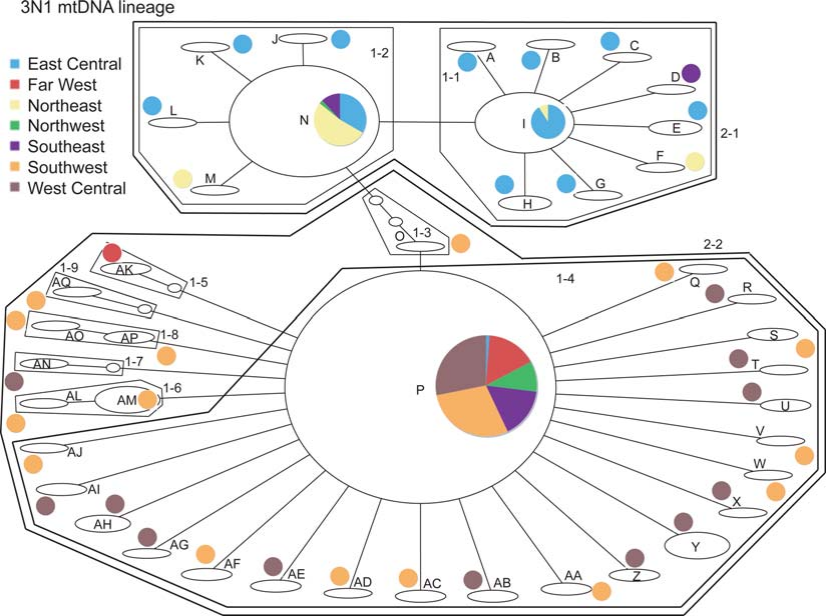
Haplotype network for the 3N1 mtDNA lineage, showing nesting levels. Clades correspond to those listed in Table 2. Small ovals without letter names are haplotypes not sampled but which are necessary to connect sampled haplotypes. Pie charts next to each haplotype indicate the proportion of individuals with that haplotype sampled from the various regions described in the analytical methods and Figure 7.

**Figure 2:**
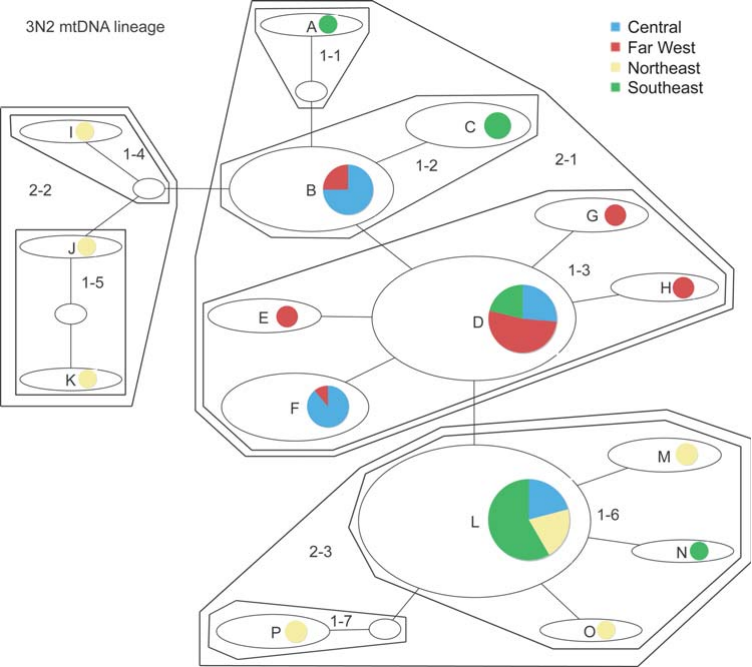
Haplotype network for the 3N2 mtDNA lineage, showing nesting levels. Clades correspond to those listed in Table 2. Small ovals without letter names are haplotypes not sampled but which are necessary to connect sampled haplotypes. Pie charts next to each haplotype indicate the proportion of individuals with that haplotype sampled from the various regions described in the analytical methods and Figure 7.

NCPA nesting clade names correspond to those in Figures 1 and 2 for the 3N1 and 3N2 lineages, respectively.

### Nested Clade Phylogeographic Analyses

Haplotype networks for the 3N1 and 3N2 mtDNA lineages are shown in Figures 1 and 2, respectively. Geographic distributions of the nesting clades are given in Table 1. In the 3N1 lineage, clade 2–1 is almost exclusively (81 of 82 individuals) restricted to eastern populations, and clade 2–2 is mostly (133 of 148 individuals) restricted to western populations. In the 3N2 lineage, clade 2–2 is restricted to the northeastern part of the range, and clade 2–1 is restricted to the rest of the range; clade 2–3 occurs in all but the Far West region. Significant clade and nested clade distances and NCPA inferences for the two lineages are given in Table 2. Clades with nonsignificant distance values or for which the interpretation was ambiguous are not included.

**Table 2:** Results of NCPA for each mtDNA lineage.

Clone	Clade	Chain of Inference	Result	Age (MYA)	C.I. (MYA)
3N1	1-1	1-2-3-5-6-TOO FEW-7-YES	DRD in eastern part of range, range expansion to southeast	0.06	0.001–0.23
	1-2	1-2-3-4-NO	DRD in east/central part of range		
	1-4	1-2-3-4-NO	DRD in western and central parts of range		
	2-1	1-2-3-4-NO	DRD in eastern part of range		
	2-2	1-2-3-4-NO	DRD in western and central parts of range		
	Total	1-19-20-2-11-12-13-YES	Range expansion from west to east, possibly followed by some fragmentation between east and west	0.24	0.025–0.73
	-	-	Age based on divergence from CA6 mtDNA	2.65	0.54–6.67
3N2	1-2	1-2-3-4-NO	DRD in west and central		
	1-3	1-2-11-12-NO	Range expansion from west/central to north/west parts of range	0.06	0.001–0.23
	1-6	1-2-11-12-NO	Range expansion from south/central to north/east parts of range	0.06	0.001–0.23
	2-1	1-2-3-4-NO	DRD throughout most of range		
	2-3	1-2-3-4-NO	DRD in central, northern, and eastern parts of range		
	Total	1-2-11-12-NO	Range expansion from west/central to north/east parts of range	0.07	0.006-0.22
	-	-	Age based on divergence from SM6 mtDNA	1.21	0.21-3.20

Only clades with significant values are shown. DRD = dispersal restricted by distance. For dates and confidence intervals, point estimates are based on an estimate of 1.3% sequence divergence per million years for this portion of the mtDNA genome. 95% Confidence intervals are based on a gamma probability distribution for coalescence time and a range of 1.22-1.4% divergence per million years (see methods). MYA = million years ago.

For the 3N1 lineage, most inferences at low and intermediate nesting levels are dispersal restricted by distance. There is evidence for recent range expansion into the narrow southeastern portion of its range, where this lineage coexists with EA6 sexuals. The oldest inference is a range expansion from west to east, corresponding to the initial expansion following their formation in the west via hybridization between the CA6 and SM6 sexuals. This event is dated at 0.24 MYA (range 0.025–0.73 MYA). The fact that lower and intermediate nesting levels have signatures of dispersal restricted by distance, including in the central and eastern parts of the range, suggests that any continuing range expansion is relatively slow. However, it does still appear to be occurring at the eastern edge of the range, as evidenced by the inference of range expansion to the southeast in clade 1–1 (Table 2). There is also evidence at the highest nesting level for fragmentation between eastern and western 3N1 populations.

In the 3N2 lineage, there are inferences of dispersal restricted by distance at both lower and intermediate nesting levels. However, there are also several inferences of range expansion at multiple nesting levels. These inferences suggest that 3N2 parthenogens were formed in roughly the southern or western portion of their current range approximately 0.07 MYA (range 0.006–0.22 MYA) and have been spreading, and are continuing to spread, to the north and east.

Dates for origins of the 3N1 and 3N2 lineages based solely on their mtDNA divergence from the mostly closely related sampled CA6 and SM6 mtDNA haplotypes, respectively, are 2.65 MYA for 3N1's (range 0.54–6.67 MYA) and 1.21 MYA for 3N2's (range 0.21–3.20 MYA), suggesting that they are older than their respective earliest NCPA inferences. Dates for NCPA-inferred initial range expansions are lower bounds for the ages of each event, because dating is based on the *youngest* monophyletic clade of the haplotype network for which the inference of range expansion applies [20]. However, the limited mtDNA diversity within each lineage makes it very unlikely that they are as old as their divergence from related sexual haplotypes would suggest. A selective sweep within each group is an unlikely explanation for this limited diversity because nuclear backcross clonal diversity is much higher (16, and Strasburg and Kearney in prep), and since the cytoplasmic and nuclear genomes are in complete linkage in these parthenogens, a sweep in one would affect the other as well. The most likely explanation is that more closely related sexual haplotypes were not sampled. This is a plausible explanation because regional divergence among CA6 and SM6 mtDNA populations can be as much as 4–5% and 7–8%, respectively [29]. Minimum divergence between 3N1 and CA6 haplotypes is 4–5%, and between 3N2 and SM6 haplotypes it is 2–3%.

### Effective Migration

The validity of NCPA for inferring population structure and historical events has been questioned [32], [33]. Although many of these criticisms have been rebutted [34], the inherent uncertainty in any such analysis warrants multiple alternative methods of inference. Consequently, we have also implemented coalescent-based analyses of effective migration in addition to more traditional distance-based analyses.

Results from coalescent-based migration analyses are shown in Table 3. Effective migration rate estimates generally had very large confidence intervals, with lower ends of those intervals often far below 0.1, suggesting that a high degree of subdivision among these particular regions cannot be rejected. Only effective migration rate estimates with confidence intervals completely above 0.1 are considered significant.

**Table 3:** Effective migration rates (average number of effective migrants per generation) among regions within each mtDNA lineage.

Clone/Source Region	Receiving Region						
3N1	East Central	Far West	Northeast	Northwest	Southeast	Southwest	West Central
East Central	-	0–0.14	***0.42–2.13***	0–0.07	0.05–4.06	0–0.65	0–0.55
Far West	0–0.17	-	0–0.34	0.05–0.21	***0.69–7.17***	***4.99–11.36***	0–0.55
Northeast	0–0.17	0–0.14	-	0–0.03	***1.15–8.57***	0–0.65	0–0.55
Northwest	0–0.17	***1.64–3.30***	0–0.34	-	***5.93–18.60***	0–0.65	***3.55–8.57***
Southeast	0–0.17	0–0.14	0–0.34	0.03–0.17	-	0–0.65	0–0.55
Southwest	0.01–0.39	0–0.14	0–0.34	0–0.03	0–1.77	-	0–0.55
West Central	0–0.17	0–0.14	0–0.34	0–0.03	0.05–4.06	0–0.65	-
3N2	Central	Far West	Northeast	Southeast			
Central	-	***1.27–4.19***	0–0.68	0–0.22			
Far West	0–0.19	-	0–0.68	***0.14–1.06***			
Northeast	0	0	-	0			
Southeast	0.03–0.51	0–0.43	***1.06–4.79***	-			

Values shown are 95% confidence intervals for N_ef_m = effective migration rate = inbreeding effective population size times proportion of individuals migrating. Direction of migration is from the region listed at left to the region listed across the top. Significant migration events (defined as those estimates whose confidence intervals are completely above 0.1) are shown in bold italics.

Effective migration results for 3N1 strongly support NCPA inferences of formation in the western portion of the range and spread to the east and southeast. All significant migration occurs within the western portion of the range or from west to east; no migration was inferred out of the southeast or from east to west. This highly asymmetric migration includes significant migration inferred from most other regions, and from the Northwest region in particular, to the Southeast region, where NCPA inferred a recent and possibly continuing range expansion event.

While it is clear from NCPA and from phylogenetic relationships between 3N1 and CA6 haplotypes that 3N1 parthenogens originated in the western portion of their range, neither analysis offers a more precise estimate of location. These migration analyses suggest that the most likely location of origin is the northern part of the western portion of the range (the Northwest region). There has been asymmetric migration from this region to the far western part of the range, and to the east and southeast, with no evidence of significant migration into the Northwest region.

Migration analysis of 3N2 is also concordant with NCPA inferences, which suggested an origin in the southern or western portion of their range and subsequent spread to the north and east. There has been significant migration from western regions to the southeast, and migration from the southeast to the northeast.

### Mismatch Distributions, Analyses of Molecular Variance

Other evidence for population growth can be obtained from an examination of the distributions of pairwise differences among haplotypes, or mismatch distributions [35], [36]. Populations that have undergone or are undergoing periods of growth tend to have a unimodal distribution of pairwise differences, with the mode shifting to the right with time following growth. Conversely, stable populations tend to show multimodal “ragged” mismatch distributions [37].

**Figure 3:**
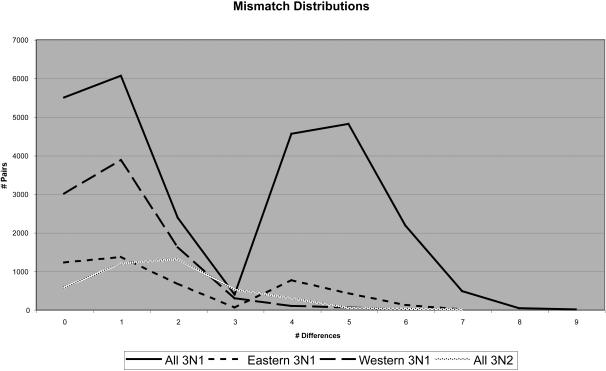
Mismatch distributions for 3N1 and 3N2 lineages. Distributions are shown for all 3N1 or 3N2 populations together and for eastern and western 3N1 populations.

Graphs of mismatch distributions for each mtDNA lineage, and for eastern and western portions of the 3N1 range, are shown in Figure 3. In each case, the distribution is clearly unimodal or bimodal. The 3N2 clone has a strongly unimodal mismatch distribution; the estimate of τ, time since expansion measured in units of 1/(2u) generations [where u is total substitution rate over all sites–38], based on the least-squares method implemented in Arlequin is 1.82, and a sudden expansion model cannot be rejected for this data. Based on our substitution rate estimate of 0.65% per lineage per million years, this corresponds to a timing of approximately 0.11 MYA for the initial expansion of 3N2's following formation. This time is very consistent with the estimate made based on the NCPA inference of northward and eastward expansion (0.07 MYA, range 0.006–0.22 MYA). In addition, estimates of Tajima's [39] D and Fu's [40] Fs were significantly negative, indicating population expansion.

The overall 3N1 distribution shows two peaks, at one and five differences. The peak at five differences corresponds to a timing of approximately 0.30 MYA, which coincides well with the timing of the initial expansion event inferred by NCPA (0.24 MYA, range 0.025–0.73 MYA). The peak at one difference corresponds to a timing of approximately 0.06 MYA, which is the same time as the estimate for range expansion to the southeast portion of the range inferred by NCPA (0.06 MYA, range 0.001–0.23 MYA). Further examination of Figure 3 reveals that this bimodality is due to eastern 3N1 individuals, while western 3N1 individuals show a unimodal mismatch distribution. This bimodality is likely the result of contraction/fragmentation during the LGM and subsequent continued expansion to the east and south.

The estimate of τ for western 3N1 is 1.04, corresponding to 0.06 MYA for this expansion-considerably more recent than estimates for eastern expansion. Based on a variety of evidence (NCPA, effective migration, affinity of 3N1 mtDNA with western CA6 mtDNA, and affinity of many 3N1 chromosomal and allozyme variants with western CA6 and SM6 variants–16), it is clear that the 3N1 mtDNA clone originated in western Australia. Therefore, this expansion in western 3N1 may also reflect Holocene expansion following contraction during the LGM. Eastern, western, and overall 3N1 fit a sudden expansion model of population growth. Tajima's D is significantly negative for western and overall 3N1 (western D = −2.38, p<0.0001; overall D = −1.81, p = 0.006), and Fu's Fs is significantly negative for all three groups (eastern Fs = −6.57, p = 0.009; western Fs<−100, p<0.0001; overall Fs = −10.65, p = 0.002).

Results from AMOVA of mtDNA for each lineage are shown in Table 4. Groups for AMOVAs are the same regions that were used for effective migration analyses. For both mtDNA lineages, among region, within region, and within population comparisons all explain a significant portion of genetic variation. However, the distribution of variation is quite different between the two lineages. Relatively little variation is distributed among regions in the 3N2 lineage, and most of the remaining variation is found within populations; this is consistent with the more recent origin of the 3N2's and their comparatively small range. In the 3N1 lineage, more than two thirds of the variation is distributed among regions. However, in an AMOVA with eastern and western populations as the groups, an almost identical amount of variation (66.5%) is distributed between groups, suggesting that almost all of this regional variation is distributed between eastern and western populations. This is consistent with the NCPA inference of a possible relatively old fragmentation event between eastern and western populations. Within regions, almost all variation is found within rather than among populations.

**Table 4:** AMOVA results for mtDNA sequence.

Race	Source	d. f.	Sum Sq.	% Variation	P
3N1	Among Regions	6	195.471	66.75	<0.00001
	Among Pops	34	24.751	3.30	0.00131
	Within Pops	188	85.909	29.95	<0.00001
	Total	228	306.131		
3N2	Among Regions	3	13.844	13.80	0.00875
	Among Pops	14	24.250	28.42	<0.00001
	Within Pops	70	36.474	57.78	<0.00001
	Total	87	74.568		

Mantel tests [41] of correlation between geographic distance and genetic distance were performed on each mtDNA lineage as a whole and within the 3N1 mtDNA lineage for eastern and western populations separately. For all tests, there is a significant correlation between geographic and genetic distance. In the 3N1's, the correlation was lowest (but still significant) among western populations (western 3N1 r = 0.19, p = 0.049; eastern 3N1 r = 0.47, p = 0.002; overall 3N1 r = 0.53, p<0.0001; 3N2 r = 0.30, p = 0.003). Mantel tests were also run on the same regions used in previous analyses, but almost all results were not significant even if correlation coefficients were high, most likely due to small sample sizes.

### Distribution Modeling

**Figure 4:**
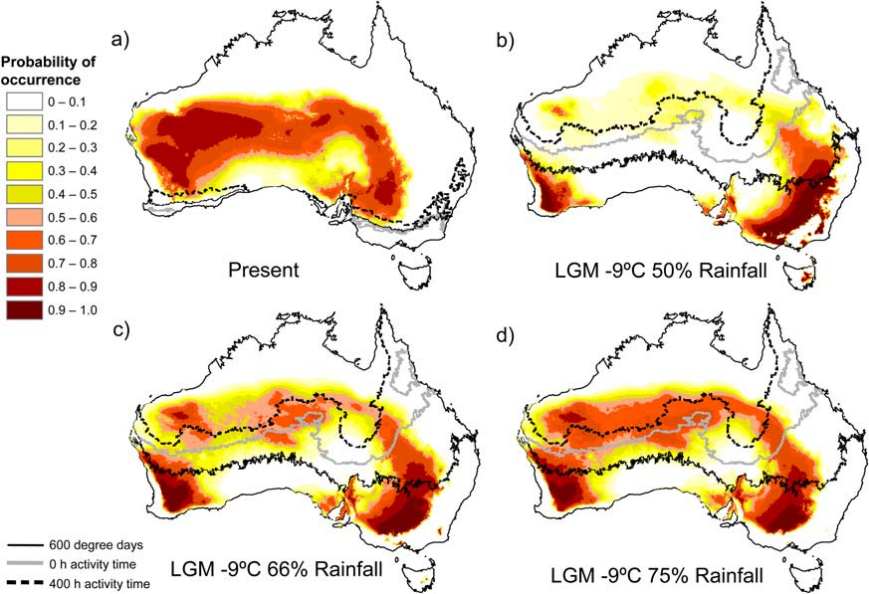
Statistical distribution models for parthenogenetic *Heteronotia binoei.* Models are for (a) present conditions and (b–d) last glacial maximum conditions with a 9°C reduction in mean air temperature and three different rainfall reduction scenarios. All statistical models are based on the AICc model reported in Kearney et al (2003). Overlayed on the predicted distributions are the contours for biophysical predictions of the limit for successful development of eggs (600 degree days), of the limit for potential activity time (0 hours) and of the number of hours of potential activity at the current distribution limit of the *Heteronotia* complex (400 hours). Any regions roughly south of the contours are outside the fundamental niche of *Heteronotia*. The biophysical predictions use either (a) current climatic conditions or (b–d) a 9°C decrease in monthly maximum and minimum temperatures. Methods for biophysical predictions are described in Kearney and Porter (2004).

Kearney *et al.* [24] found that the current distribution of parthenogenetic *H. binoei* coincides fairly closely with their expected distribution based on correlations with six temperature and rainfall variables in western Australia, while considerable similar but unoccupied habitat exists in central and eastern Australia. Taking a more mechanistic approach, Kearney and Porter [25] found that the current southern distribution of the *H. binoei* complex is partially limited by temperature requirements for successful egg development and foraging activity. Here we have applied these approaches using estimates of climatic conditions during the LGM. Average air temperatures in the interior of Australia were around 9°C cooler 16–45 KYA than at present [42]. The arid zone was also considerably drier during the LGM, although estimates of the degree of aridification vary [31]. Predicted correlative distribution models for parthenogenetic *H. binoei* and biophysical predictions for the temperature limits for successful egg developments and minimal foraging activity are shown in Figure 4. Three scenarios are presented, with mean annual rainfall reductions of 1/2, 1/3, and 1/4 (all with a 9°C average temperature reduction).

Probability of occurrence based on correlations with temperature and rainfall variables decreases dramatically throughout much of the interior of Australia under all three scenarios; probability density is shifted to southeastern and southwestern Australia, where rainfall amounts are similar to current levels in the interior. However, the 9°C temperature decrease shifts the contours for biophysical predictions of minimal temperatures for successful egg development and foraging far to the north. Under our assumptions of temperature and rainfall conditions during the LGM, and assuming that climatic correlates and biophysical requirements of current *H. binoei* are comparable to those of the LGM, the regions where they were most likely to persist during the LGM were the northwest and north-central parts of the arid zone.

## Discussion

### Phylogeographic History of *H. binoei* Parthenogens

NCPA of the 3N1 and 3N2 parthenogens reveal a recent origin of each lineage and subsequent spread to the east and south (3N1) and east and north (3N2). Dating estimates of the oldest NCPA inferences, which correspond to initial expansion following formation, put the 3N1 expansion at 0.24 MYA (range 0.025–0.73 MYA), and the 3N2 expansion at 0.07 MYA (range 0.006–0.22 MYA). As mentioned above, these dates for NCPA inferences of initial range expansion are based on the youngest monophyletic clade of the haplotype network for which the inference of range expansion applies [20], which in each case corresponds to one or more of the highest level nesting clades; thus these range expansion dates set lower bounds for the ages of each lineage. There is no evidence suggesting that there would have been any substantial delay between formation and range expansion in either lineage, and we expect dates for formation to be close to dates for initial range expansion. Analyses of effective migration support northwestern and southwestern or central-western origins for the 3N1 and 3N2 lineages, respectively. NCPA and effective migration also indicate recent and possibly ongoing expansions to the southeast in 3N1's and to the east in 3N2's, and mismatch distributions also suggest rapid population growth in each lineage. Coalescent analyses of effective population growth show that overall, and in most regions, populations of both parthenogens are growing very quickly, as would be expected under a scenario of recent and rapid range expansion (data not shown).

There is also evidence at the highest NCPA nesting level for fragmentation between eastern and western 3N1 populations. In addition, AMOVA using the eastern and western areas as groups reveals a large amount (66%) of variation distributed between groups, and eastern and western haplotypes are mostly segregated at the highest levels of nesting in the haplotype network (Figure 1). However, analyses of effective migration (Table 3) provide no evidence of east/west fragmentation; in fact, there is strong signal of west to east migration. Sampling in the middle portion of the 3N1 range is somewhat sparse in comparison to more eastern and western areas, and this may be partially responsible for an inference of fragmentation; more sampling in the region may reveal intermediate haplotypes and more continuity between east and west. While this fragmentation inference may be considered slightly tentative, it is interesting that the predicted distribution of parthenogenetic *H. binoei* during the LGM under 33% and 50% rainfall reduction scenarios is somewhat discontinuous in this region (Figure 4), with an area of low probability of occurrence, corresponding roughly with the fragmentation event, separating two areas of higher probability of occurrence (see “Distribution Modeling” below).

Based on the mtDNA restriction profiles showing an affinity of 3N2 mtDNA with a clade of SM6 haplotypes from the extreme western part of their range along the northwest coast of Australia, Moritz and Heideman [27] concluded that the 3N2 mtDNA lineage had likely originated in the northwestern part of its range (see Figure 2 in 26). Under this scenario, 3N2 parthenogens then spread to the east and south to occupy their current range. However, based on our mtDNA sequence data [29] this SM6 clade also includes a haplotype sampled from near Shark Bay at the west-central edge of the 3N2 range. No other SM6 individuals were sampled within 400 km of this population (see Figure 2, 29), so it could well be a remnant population from a more southern historical distribution of this race. It seems likely that the CA6 and/or SM6 ranges in this area have changed substantially due to Pleistocene climatic changes (see below), and these range shifts facilitated hybridization between ecologically and genetically distinct races in this group [29], [43]. This, combined with NCPA and effective migration analyses showing range expansion and movement to the east and north in the 3N2 lineage, make it most likely that the 3N2 parthenogens were formed in the west-central or southwest part of their current range.

### Distribution Modeling

**Figure 5:**
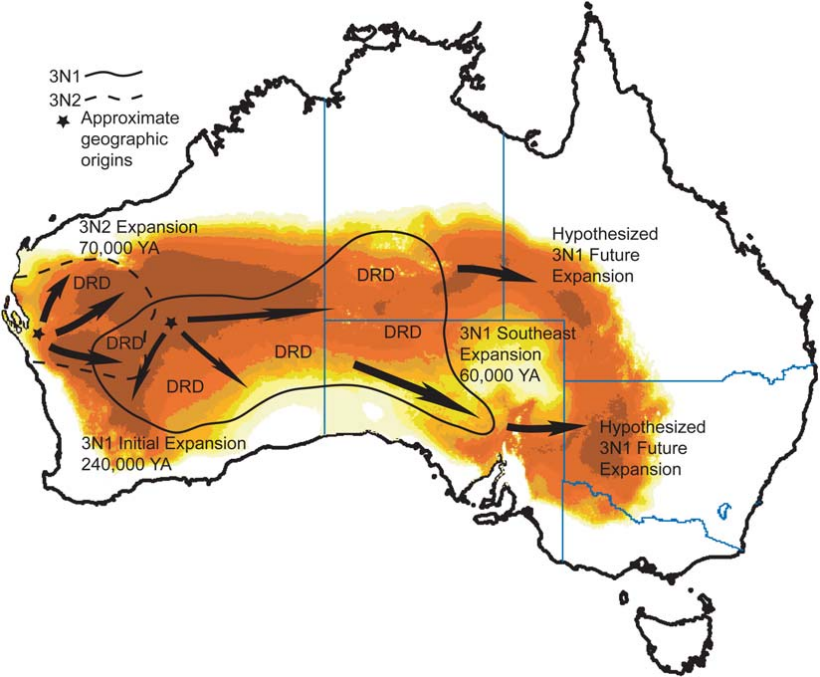
Proposed origin and spread of 3N1 and 3N2 parthenogens. Also shown are timing estimates for expansions and hypothesized future expansions in 3N1 parthenogens. Phylogeographic events are overlaid on the predicted distribution for parthenogenetic *Heteronotia binoei* based on a statistical distribution model for present climatic conditions [24]. Times given here are point estimates; confidence intervals are given in Table 2. DRD = dispersal restricted by distance.

Modeling of the climatic correlates of *H. binoei* distributions and biophysical modeling of limits for successful egg development and minimal foraging time strengthen our phylogeographic scenario. Kearney *et al.* [24] analyzed the bioclimatic envelopes of each asexual lineage and found that six climatic variables related to temperature and rainfall fairly accurately describe the distributions of each lineage in the western parts of their ranges, but that in each case large areas of climatically similar habitat exist to the east of their current ranges (see Figure 9 in 24). This result is in agreement with our inference of recent and continuing eastward expansion within each lineage. Concordance between the predicted 3N1 range based on these climatic variables and our inferred range expansion is especially striking–an uninhabitable area in the Lake Eyre basin around northeast South Australia, southeast Northern Territory, and southwest Queensland is mostly surrounded by more suitable habitat (see Figure 9 in 24), and the southern part of this circle corresponds to the recent southeastern range expansion and our predicted continuing expansions (Figure 5).

It is significant that the 3N1 lineage has not expanded further into the southwestern part of Australia, an area where no *H. binoei* exist. Kearney and Porter [25] showed that in many places the southern limit of the range of the EA6 sexual chromosome race (the most southerly distributed chromosome race) coincides very closely with the thermal limit for successful egg development; similar climatic constraints on the 3N1 southern distribution are likely to be in place.

We repeated these correlative analyses for both parthenogenetic lineages combined, under current climatic conditions as well as under three different scenarios for the LGM–a uniform 9°C decrease in average air temperature along with rainfall reductions of 25%, 33%, and 50% (Figure 4). During the LGM, rainfall conditions similar to those in present-day parthenogenetic *H. binoei* ranges would mostly have been restricted to extreme southwestern and southeastern Australia. Rainfall was strongly weighted in the correlative distribution model for parthenogenetic *Heteronotia* (Kearney et al. 2003) hence the prediction for a significant southward shift in the distribution.

We have also extended the biophysical modeling of Kearney and Porter [25] to include these LGM scenarios (overlaid contour lines on Figure 4b–d for the 600 degree days necessary for successful egg development and the zero and 400 hours potential activity time contours). Correlative distribution model predictions are discordant with those of the biophysical model, which shows that most of the areas of highest probability density in the correlative model are well south of the 600 degree day and zero hours potential activity contour lines, and so would likely have been outside the fundamental niche of *H. binoei* based on these biophysical requirements [25]. Regions of most similar habitat north of the contour lines are found in the northwest and north-central parts of the arid zone, and for the 33% and 50% rainfall reduction scenarios they are separated by an area of somewhat less similar habitat. The absence of extremely cold and arid environments in Australia at present is presumably why extrapolation of the regression model results in a biologically unrealistic prediction.

It is particularly interesting to note that the biophysical model predicts potential activity time to be more limiting than potential egg development time during glacial maxima, whereas the reverse is true under current climatic conditions. This occurs because egg development rate in the soil is affected by solar radiation and air temperature, while potential activity time in this nocturnal lizard is solely affected by air temperature. Potential activity time is more severely affected because our modeling assumes that the air temperature changes between glacial cycles but solar radiation does not. In this respect, it may be significant that parthenogenetic *H. binoei* have evolved greater aerobic endurance at low temperature when compared with their sexual relatives [44].

### Concordance between phylogeographic analyses and distribution modeling

While our modeling for the LGM is somewhat crude in that it assumes geographically uniform changes in temperature and rainfall (probably not a realistic assumption–31), it is in substantial agreement with our phylogeographic results, summarized in Figure 5. We have inferred an origin of the 3N1 mitochondrial lineage approximately 240,000 years ago, likely during the previous glacial cycle, in the northwest part of its range. This would have been near the southern limit of the fundamental niche of the *Heteronotia* complex (assuming roughly similar conditions during the glacial maximum previous to the LGM), and it is reasonable to expect that the CA6 and SM6 sexual races would have come into contact in this region as the range of each was contracted northward. Following some, mostly eastward, expansion, the 3N1 range contracted to the northwest and north-central arid zone during the LGM, possibly into two disjunct regions (see Figure 4b–d). This is a likely cause of the fragmentation event inferred at higher levels of nesting in the 3N1 NCPA. Also during the LGM, the 3N2 parthenogens were formed via a second period of contact and hybridization between the CA6 and SM6 races in Western Australia. Under this scenario, the range of the SM6 sexuals during the LGM extended further to the south in this area, and the population from Shark Bay is a remnant of this southern range.

Results for both 3N1 and 3N2 lineages suggest that abiotic factors may play the most important role in determining their geographic distributions. However, it is worth pointing out that both lineages appear to still be expanding their ranges, and so are likely in a non-equilibrium state. In addition, Moritz *et al.* [45] found much higher rates of infection by parasitic mites for parthenogenetic *H. binoei* sampled throughout their range relative to their sexual counterparts. Studies of the environmental and physiological tolerances of different parthenogenetic clones are underway (Kearney and Strasburg in prep), and further studies involving direct competition and transplant experiments will help strengthen inferences of limiting factors in parthenogen distributions.

The Australian arid zone is home to a diverse array of hybrid parthenogens [reviewed in 46], and those that have been studied in detail also appear to have late Pleistocene origins [30], [47]. Many explanations have been put forth for the persistence of parthenogens in the arid zone and elsewhere [9], [10], [48]–[50], and the role of climatic cycling in hybridization is well-documented [51]. It may be the case that similar climatic conditions have driven the hybridization events resulting in other arid zone parthenogens, and that similar factors constrain their distributions. We were able to make robust inferences about the history of the *H. binoei* complex in relation to climatic cycles by combining population genetic approaches with climatic and biophysical distribution modeling. This methodology should also be very valuable for understanding the prevalence of hybrid parthenogenesis in the Australian arid zone, and for addressing the role of abiotic factors in the formation, spread, and persistence of parthenogenetic lineages more generally.

## Materials and Methods

### Field

**Figure 6:**
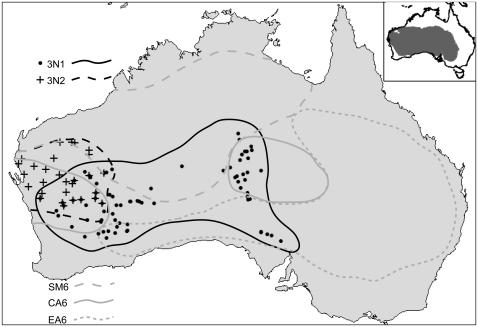
Sampling localities for the 3N1 and 3N2 mitochondrial clones. Latitude/longitude data and sample sizes are given in Table 5. Ranges of the CA6, EA6, and SM6 sexual chromosome races are shown in light gray. The inset in the upper right shows the extent of the arid zone in dark gray.

Our analyses are based on 319 specimens of parthenogenetic *H. binoei*, encompassing the ranges of the two mtDNA lineages known as 3N1 and 3N2. Of these samples, 127 were collected in the 1980's and early 1990's [26] and 192 were collected in 2000–2001 (Table 5 and Figure 6). In some cases, nearby populations with small population sizes were combined for analyses. For the 2001 collections, representative individuals from each population were euthanized for voucher specimens, and for the rest tail tips were taken and the individuals were released. Voucher specimens are deposited in the South Australian Museum, Australian National Wildlife Collection, Queensland Museum, and University of Michigan Museum of Zoology (for individuals collected by C. Moritz), and in the Western Australian Museum (for individuals collected in 2001). Museum catalog numbers for voucher specimens are given in Table 5.

**Table 5:** Sampling localities, sample sizes, and mtDNA haplotypes sampled at each locality.

			mtDNA Lineage				
Locality	Latitude	Longitude	3N1	3N2	Museum Catalog Numbers	GenBank Accession Numbers	Haplotypes
17kms S Leonora WA	−29.033	121.317	1		ABTC32512	DQ287414	3N1-P
22km NE Bonnie Rock WA	−30.383	118.500	1		ABTC31222	DQ287372	3N1-P
4km W Johnston Rocks WA	−29.800	119.833	1		ABTC31220	DQ287371	3N1-P
Aileron NT	−22.648	133.352	5			DQ287444, DQ287463, DQ287470, DQ287473, DQ287475	3N1-N (5)
Alice Springs NT	−23.700	133.867	2		ABTC32565, ABTC32566	DQ287419, DQ287420	3N1-N (2)
Avoca Downs Station	−30.950	122.318	4		R144987	DQ287495, DQ287544, DQ287550, DQ287589	3N1-AD (2), 3N1-AM (2)
Bandya WA	−27.550	121.828	7		R146801	DQ287496, DQ287498, DQ287516, DQ287521, DQ287531, DQ287545, DQ287571	3N1-P (2), 3N1-T, 3N1-Y, 3N1-Z (2), 3N1-AN
Belele WA	−26.367	118.017		5	ABTC32361, ABTC32373, ABTC32445, ABTC32457, ABTC32461	DQ287604, DQ287608, DQ287626, DQ287632, DQ287633	3N2-B (2), 3N2-D (3)
Billabalong WA	−27.417	115.833		3	ABTC32371, ABTC32374, ABTC32385	DQ287607, DQ287609, DQ287613	3N2-D (2), 3N2-E
Billabong WA	−26.817	114.617		4	ABTC32444, ABTC32526, ABTC32530, ABTC32542	DQ287625, DQ287641, DQ287643, DQ287647	3N2-D (2), 3N2-E (2)
Boologooroo WA	−24.333	114.033		2	ABTC32378, ABTC32379	DQ287610, DQ287611	3N2-H (2)
Brickhouse WA	−24.817	113.783		5	ABTC32796, ABTC32800, ABTC32916, ABTC33076, ABTC33078	DQ287655, DQ287656, DQ287667, DQ287673, DQ287674	3N2-B (2), 3N2-G (3)
Bulga Downs WA	−28.494	119.739	5		R146770	DQ287560, DQ287563, DQ287570, DQ287575, DQ287576	3N1-P (5)
Bullabulling WA	−31.017	120.867	3		ABTC32364, ABTC32470, ABTC32490	DQ287385, DQ287403, DQ287411	3N1-P, 3N1-AC (2)
Bullardoo Stn WA	−27.850	115.667	2	2	ABTC32458, ABTC32471, ABTC32386, ABTC32447	DQ287400, DQ287404, DQ287614, DQ287628	3N1-P (2), 3N2-D (2)
Bundarra Stn WA	−28.317	121.167	2	2	ABTC32504, ABTC32534, ABTC32507, ABTC32537	DQ287413, DQ287416, DQ287637, DQ287645	3N1-AB (2), 3N2-L (2)
Coondambo SA	−31.060	135.865	3		ABTC32440	DQ287398, DQ287456, DQ287488	3N1-P (3)
Copper Hill SA	−27.950	134.313	1			DQ287446	3N1-A
Cundeelee WA	−30.720	123.422	11		R146757, R146792	DQ287494, DQ287517, DQ287526, DQ287553, DQ287557, DQ287558, DQ287569, DQ287572, DQ287586, DQ287587, DQ287597	3N1-P (5), 3N1-AA (2), 3N1-AM (4)
Cunyu WA	−26.017	120.117	1		ABTC32404	DQ287387	3N1-P
Curbur WA	−26.467	115.933		4	ABTC32354, ABTC32446, ABTC32450, ABTC32528	DQ287602, DQ287627, DQ287630, DQ287642	3N2-D (3), 3N2-F
Dalgetty Downs WA	−25.283	116.200		5	ABTC32370, ABTC32387, ABTC33128, ABTC33130, ABTC33131	DQ287606, DQ287615, DQ287675, DQ287676, DQ287677	3N2-D (2), 3N2-F (3)
De Rose Hill SA	−26.417	133.250	2		ABTC32431, ABTC32522	DQ287394, DQ287415	3N1-I, 3N1-L
Deep Well NT	−24.298	134.133	4			DQ287465, DQ287468, DQ287471, DQ287600	3N1-I, 3N1-N (3)
Earaheedy WA	−25.550	121.583	1		ABTC32397	DQ287386	3N1-P
East edge of Yeo Lake WA	−28.236	124.673	1			DQ287542	3N1-P
East of Yeo WA	−28.136	124.465	7		R146769, R146781, R146803, R146804, R146808	DQ287504, DQ287512, DQ287537, DQ287549, DQ287564, DQ287581, DQ287596	3N1-P (3), 3N1-Y (4)
Glenayle WA	−25.267	122.033	1		ABTC31426	DQ287381	3N1-P
Goongarrie WA	−29.984	121.044	6		R145049	DQ287497, DQ287503, DQ287515, DQ287533, DQ287574, DQ287585	3N1-P (3), 3N1-AF (2), 3N1-AP
Granite Downs SA	−26.937	133.495	5		ABTC32435, ABTC32486	DQ287396, DQ287409, DQ287452, DQ287486, DQ287490	3N1-E, 3N1-I, 3N1-N (3)
Granite Peak WA	−25.633	121.350		1	ABTC32541	DQ287646	3N2-N
Hampton Hill WA	−30.762	121.737	4		R146787	DQ287519, DQ287556, DQ287598, DQ287599	3N1-S (2), 3N1-AO, 3N1-AP
Jimba Jimba WA	−25.033	115.133		1	ABTC32930	DQ287670	3N2-B
Juna Downs WA	−22.883	118.483		4	ABTC32773, ABTC32819, ABTC32820, ABTC32834	DQ287652, DQ287659, DQ287660, DQ287662	3N2-L (3), 3N2-O
Kathleen Valley WA	−27.400	120.650		1	ABTC32509	DQ287638	3N2-L
Kingoonya SA	−30.912	135.315	2			DQ287437, DQ287482	3N1-P (2)
Kirkalocka WA	−28.555	117.777	1	2	ABTC32408, ABTC32417, ABTC32525	DQ287389, DQ287622, DQ287640	3N1-P, 3N2-L (2)
Lake Violet WA	−26.533	120.667	2		ABTC32418, ABTC32421	DQ287390, DQ287392	3N1-P (2)
Leinster Downs WA	−27.850	120.600		3	ABTC32349, ABTC32426, ABTC32505	DQ287601, DQ287624, DQ287636	3N2-L (3)
Lilla Ck. NT	−25.567	134.067	1		ABTC31643	DQ287383	3N1-I
Mandilla WA	−31.376	121.537	1			DQ287566	3N1-AJ
Mt Augusta WA	−24.300	116.917		4	ABTC32847, ABTC32929, ABTC32932, ABTC32934	DQ287665, DQ287669, DQ287671, DQ287672	3N2-F (4)
Mt Cavenagh NT	−25.915	133.133	5			DQ287432, DQ287433, DQ287443, DQ287462, DQ287476	3N1-I (5)
Mt Ebenezer NT	−25.100	132.567	1		ABTC31384	DQ287378	3N1-J
Mt Gould WA	−25.800	117.383		4	ABTC32358, ABTC32367, ABTC32390, ABTC32449	DQ287603, DQ287605, DQ287617, DQ287629	3N2-B (3), 3N2-F
Mt Willoughby SA	−27.958	134.145	7		ABTC32436, ABTC32441, ABTC32469	DQ287397, DQ287399, DQ287402, DQ287449, DQ287454, DQ287458, DQ287474	3N1-E, 3N1-H (3), 3N1-I, 3N1-N, 3N1-P
Munarra WA	−26.283	118.700	2	4	ABTC31373, ABTC32472, ABTC32409, ABTC32413, ABTC32416, ABTC32477	DQ287376, DQ287405, DQ287618, DQ287620, DQ287621, DQ287635	3N1-N, 3N1-P, 3N2-B (4)
Nallan WA	−27.317	117.967		4	ABTC32388, ABTC32412, ABTC32422, ABTC32467	DQ287616, DQ287619, DQ287623, DQ287634	3N2-C, 3N2-D (3)
NE of Yamarna WA	−28.127	123.699	4		R146760	DQ287508, DQ287530, DQ287538, DQ287562	3N1-P, 3N1-Y (3)
Neale Junction WA	−28.304	125.816	1		R146765	DQ287541	3N1-U
Neds Creek WA	−25.483	119.650	1		ABTC32432	DQ287395	3N1-P
New Springs WA	−25.817	120.000	2		ABTC32485, ABTC32544	DQ287408, DQ287418	3N1-P (2)
Ninghan WA	−29.431	117.287	2			DQ287582, DQ287583	3N1-AK (2)
North Well SA	−30.843	135.310	6			DQ287424, DQ287429, DQ287445, DQ287447, DQ287453, DQ287492	3N1-D, 3N1-N (2), 3N1-P (3)
Oakden Hills SA	−31.667	137.033	3		ABTC32427, ABTC32473, ABTC32535	DQ287393, DQ287406, DQ287417	3N1-P (3)
Old Andado NT	−25.383	135.283	2		ABTC31374, ABTC31417	DQ287377, DQ287380	3N1-I, 3N1-N
Old Bandya WA	−27.697	122.134	6		R146761	DQ287501, DQ287502, DQ287511, DQ287532, DQ287535, DQ287539	3N1-P (5), 3N1-AG
Orange Ck. #1 NT	−24.450	133.450	1		ABTC31179	DQ287370	3N1-M
Orange Ck. #2 NT	−23.997	133.592	5			DQ287440, DQ287448, DQ287455, DQ287459, DQ287467	3N1-F, 3N1-N (4)
Pack Saddle Camp WA	−22.917	118.750		1	ABTC32839	DQ287663	3N2-L
Parallel Road #2 WA	−26.998	125.545	2		R145133, R146785	DQ287559, DQ287584	3N1-AI (2)
Pinjin WA	−30.080	122.732	6		R145007, R145086, R145140	DQ287518, DQ287523, DQ287524, DQ287529, DQ287543, DQ287548	3N1-P (4), 3N1-V, 3N1-AM
Queen Victoria Springs WA	−30.790	123.363	1		R146783	DQ287578	3N1-AM
Rocklea Stn WA	−22.883	117.450		4	ABTC32771, ABTC32772, ABTC32774, ABTC32817	DQ287650, DQ287651, DQ287653, DQ287657	3N2-L, 3N2-M (3)
Ross River NT	−23.597	134.485	2			DQ287421, DQ287426	3N1-I, 3N1-N
Sandstone WA	−27.992	119.292	3	1	R146762, R146777	DQ287500, DQ287520, DQ287536, DQ287678	3N1-P (3), 3N2-L
Sherwood WA	−26.567	118.533		4	ABTC32381, ABTC32453, ABTC32511, ABTC32532	DQ287612, DQ287631, DQ287639, DQ287644	3N2-L (4)
Sylvania WA	−23.583	120.050		1	ABTC32770	DQ287649	3N2-I
The Garden NT	−23.282	134.417	4			DQ287477, DQ287479, DQ287481, DQ287485	3N1-N (4)
Thundelarra WA	−28.967	117.117	1		ABTC31352	DQ287375	3N1-P
Ti Tree NT	−22.132	133.267	3			DQ287425, DQ287480, DQ287489	3N1-N (3)
Tieyon SA	−26.208	133.855	4			DQ287435, DQ287442, DQ287461, DQ287487	3N1-E, 3N1-I, 3N1-N (2)
Uluru NT	−25.417	131.967	1		ABTC31233	DQ287373	3N1-N
Umbeara NT	−25.748	133.683	5			DQ287430, DQ287450, DQ287469, DQ287472, DQ287483	3N1-G, 3N1-I (2), 3N1-K, 3N1-N
Uranerz Camp WA	−30.159	123.443	1		R146782	DQ287522	3N1-W
Victory Downs SA	−25.988	132.970	5			DQ287423, DQ287457, DQ287460, DQ287464, DQ287491	3N1-I (4), 3N1-N
Warakurna WA	−25.033	128.250	1		ABTC31392	DQ287379	3N1-N
Welbourn Hill SA	−27.357	134.085	6		ABTC31653	DQ287384, DQ287422, DQ287427, DQ287438, DQ287441, DQ287451	3N1-I (2), 3N1-N (4)
Wheelerrana Stn WA	−23.983	120.000		5	ABTC32769, ABTC32778, ABTC32818, ABTC32821, ABTC32925	DQ287648, DQ287654, DQ287658, DQ287661, DQ287668	3N2-J, 3N2-K, 3N2-P (3)
White Cliffs WA	−28.479	122.803	10		R145139, R146802	DQ287510, DQ287513, DQ287525, DQ287540, DQ287546, DQ287552, DQ287555, DQ287567, DQ287591, DQ287595	3N1-P (5), 3N1-X, 3N1-AE (4)
Windsor WA	−28.012	118.580	2	5	R146775	DQ287514, DQ287534, DQ287683, DQ287684, DQ287685, DQ287688, DQ287689	3N1-P (2), 3N2-L (5)
Wintinna SA	−27.712	134.115	3			DQ287434, DQ287466, DQ287484	3N1-B, 3N1-C, 3N1-I
Wirraminna SA	−31.190	136.228	7		ABTC31429, ABTC32495	DQ287382, DQ287412, DQ287428, DQ287431, DQ287436, DQ287439, DQ287478	3N1-N (3), 3N1-P (4)
Woomarel WA	−25.733	114.283		1	ABTC32849	DQ287666	3N2-D
Yamarna WA	−28.161	123.668	4		R145083, R146771, R146784, R146800	DQ287493, DQ287547, DQ287551, DQ287565	3N1-P, 3N1-R, 3N1-U, 3N1-AH
Yarri WA	−29.777	122.359	7		R145005, R145061	DQ287505, DQ287528, DQ287573, DQ287577, DQ287580, DQ287592, DQ287594	3N1-O, 3N1-P (5), 3N1-AL
Yellowdine WA	−31.300	119.650	1		ABTC32488	DQ287410	3N1-P
Yeo WA	−28.077	124.318	5		R145047, R146766, R146772, R146791, R146796	DQ287499, DQ287509, DQ287527, DQ287590, DQ287593	3N1-P (5)
Yindi WA	−30.384	122.507	6		R145006, R145059	DQ287506, DQ287507, DQ287554, DQ287568, DQ287579, DQ287588	3N1-P (5), 3N1-Q
Yoothapinna WA	−26.533	118.517		1	ABTC32846	DQ287664	3N2-L
Yowergabbie WA	−28.242	117.661	1	6		DQ287561, DQ287679, DQ287680, DQ287681, DQ287682, DQ287686, DQ287687	3N1-P, 3N2-A, 3N2-C (4), 3N2-D
Yundamindra WA	−29.250	122.100	2		ABTC32407, ABTC32480	DQ287388, DQ287407	3N1-P (2)
Yunndaga WA	−29.783	121.150	2		ABTC32420, ABTC32466	DQ287391, DQ287401	3N1-P, 3N1-AQ
TOTALS			230	89			

Haplotype names correspond to those in Figures 1 and 2. Sampling sites are shown in Figure 6. Museum catalog numbers beginning with ABTC refer to South Australian Museum, and those beginning with R refer to Western Australian Museum.

### Molecular

Techniques for DNA extraction, amplification and sequencing are described in Strasburg and Kearney [29]. We sequenced the *ND2* (NADH dehydrogenase subunit two) gene and flanking tRNA genes, a region particularly useful for intraspecific and intrageneric studies because of its relatively high rate of evolution [52], [53]. All sequences have been submitted to GenBank, and accession numbers are given in Table 5.

### Analytical

AMOVAs were performed for mtDNA sequence for both lineages using the computer program Arlequin 2.001 [54]. Uncorrected pairwise differences were used as the distance measure, and significance was assessed with 16,000 permutations. Mantel tests and mismatch analyses were also performed in Arlequin, with 10,000 permutations for Mantel tests and 1000 bootstrap replicates for mismatch analyses. Nucleotide diversities were calculated using Mega 2.1 [55], with standard errors calculated using the bootstrap method with 1,000 resamples.

Nested clade phylogeographic analysis (NCPA–34) was performed separately on the 3N1 and 3N2 lineages. Haplotype networks were inferred under the criterion of statistical parsimony [56] using TCS 1.16 [57], and permutations and significance tests were performed using Geodis 2.0 [58] with 10,000 permutations. Single-locus phylogeographic studies are typically limited by the fact that they cannot account for inter-locus variability due to both mutational and coalescent stochasticity. While we acknowledge the former limitation with this study, the latter is not an issue here because these geckos reproduce clonally.

Dating of NCPA inferences was performed using the method of Templeton [20]. This method allows for calculation of a point estimate for the age of a given event, and a confidence interval around that estimate that accounts for evolutionary stochasticity by modeling the distribution of time to coalescence as a gamma function [59]. Point estimates were obtained by comparing sequence diversity in the youngest monophyletic clade of the haplotype network for which the inference applies and sequence divergence from the nearest clade: divergence time t = (Dxy-0.5*(Dx+Dy))*substitution rate, where Dxy is average divergence between the focal clade and its neighboring clade, and Dx and Dy are average diversity within each clade [60]. For the section of mtDNA sequenced here, Macey *et al.* [61] estimated the rate of evolution in Agamid lizards to be 0.65% per lineage per million years (range based on geological dating estimates 0.61–0.70%), corresponding to a divergence rate of 1.3% per million years. Other studies have found highly concordant rates in other reptile, amphibian, and fish taxa [62]. In our 95% confidence intervals, we used a range of 0.61–0.70% per lineage per million years (corresponding to 1.22–1.4% divergence per million years) to account for some error in the estimate of evolutionary rate. In order to verify our assumption of equal rates of evolution along lineages for NCPA dating, a likelihood ratio test of a molecular clock [63] was performed on a tree of all sexual and parthenogenetic *H. binoei* haplotypes (including the EA6 sexual chromosome race) rooted with a single *H. planiceps* haplotype. We were unable to reject a molecular clock (2δ = 285.234, df = 255, p = 0.094; for details on maximum likelihood analysis conditions see ref. 29).

**Figure 7:**
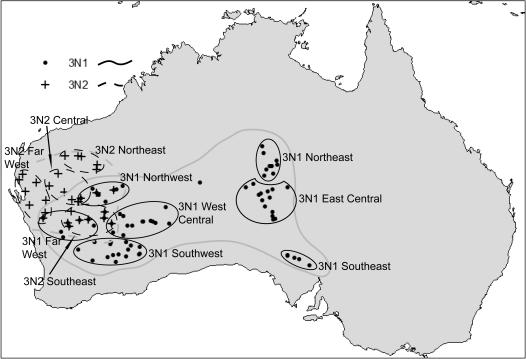
Regions used for various analyses. Ranges for each mtDNA lineage as a whole are shown in light gray.

Effective migration rates among populations and regions within each race were measured using the computer program Migrate 1.7.6 [64]. Migrate uses a Markov chain Monte Carlo approach with importance sampling [65] to estimate N_ef_m, where N_ef_ is the long-term inbreeding effective size and m is the average proportion of individuals migrating per generation. Analyses were run with 20 short chains with 1,000,000 genealogies sampled and 10,000 genealogies recorded, and 2 long chains with 10,000,000 genealogies sampled and 100,000 genealogies recorded. Analyses in Migrate were run both with individual populations and with nearby populations combined into regions to increase sample sizes and for ease of interpretation. 3N1 populations were grouped into Far West, Northwest, Southwest, West Central, East Central, Northeast, and Southeast regions, and 3N2 populations were grouped into Far West, Central, Northeast, and Southeast regions (Figure 7). Populations were grouped by eye, and in a few cases populations that were distant from any others were not included in a region. Combining populations that may show some genetic structure violates an assumption of the models underlying the coalescent techniques used in these programs; however, this is often a reasonable step to facilitate computation and interpretation of analyses [66]. Summed results from individual populations were very consistent with results from regions, suggesting that the analyses are in fact quite robust to violations of this assumption.

### Distribution modeling

We used two contrasting approaches to predict the distribution of parthenogenetic *H. binoei* during current and LGM conditions: a correlative approach and a mechanistic approach. The correlative approach was based on a previously generated logistic regression model using six climatic predictor variables including mean annual temperature rainfall and humidity, as well as temperature and rainfall variability [24]. Predictions were made using current climatic conditions, as reported in Kearney *et al.* [24], as well as estimated conditions during the LGM. These estimates involve a 9°C reduction in mean annual air temperature [42] and three scenarios of reduced mean annual rainfall (3/4, 2/3 and 1/2 of current rainfall levels), with the other four climatic variables held constant. We used a range of rainfall reduction scenarios because there is considerable uncertainty in this respect (31 and P. Hope pers. comm.).

The mechanistic approach involved applying biophysical models to predict regions where egg development and above-ground activity are possible. This approach provides a means to map the fundamental niche of an organism (see [25] and [67] for details). Previous research has shown that *H. binoei* require approximately 600 degree days above 20°C for successful egg development, and that these lizards rarely forage at air temperatures below 15°C. Biophysical predictions were made using current climatic conditions, as reported in Kearney and Porter (2004), as well as an inferred 9°C reduction in monthly maximum and minimum air temperature during the LGM [42].

We assume here that habitat preferences for parthenogenetic *H. binoei* have not changed significantly since the LGM. While there are physiological differences between parthenogenetic and sexual *H. binoei* [44] which may have been a consequence of hybridization or evolved post-hybridization, there are no obvious differences in how they use microhabitats–both shelter and lay their eggs under a wide variety of surface debris as well as in burrows.
